# Impact of multi-heavy metal exposure on renal damage indicators in Korea: An analysis using Bayesian Kernel Machine Regression

**DOI:** 10.1097/MD.0000000000035001

**Published:** 2023-10-13

**Authors:** Sun-Haeng Choi, Kyung Hi Choi, Jong-Uk Won, Heon Kim

**Affiliations:** a Department of Occupational and Environmental Medicine, Chungbuk National University Hospital, Cheongju, Republic of Korea; b Department of Public Health, Graduate School, Yonsei University, Seoul, Republic of Korea; c Department of Preventive Medicine, College of Medicine, Chungbuk National University, Cheongju, Republic of Korea.

**Keywords:** BKMR analysis, heavy metals, refinery, renal tubular damage

## Abstract

Exposure to cadmium (Cd), arsenic (As), and mercury (Hg) is associated with renal tubular damage. People living near refineries are often exposed to multiple heavy metals at high concentrations. This cross-sectional study investigated the association between combined urinary Cd, As, and Hg levels and renal damage markers in 871 residents living near the Janghang refinery plant and in a control area. Urinary Cd, As, Hg, N-acetyl-β-D-glucosaminidase (NAG), and β2-microglobulin (β2-MG) levels were measured. The combined effects of Cd, As, and Hg on renal tubular damage markers were assessed using linear regression and a Bayesian Kernel Machine Regression (BKMR) model. The results of the BKMR model were compared using a stratified analysis of the exposure and control groups. While the linear regression showed that only Cd concentration was significantly associated with urinary NAG levels (β = 0.447, *P* value < .05), the BKMR model showed that Cd and Hg levels were also significantly associated with urinary NAG levels. The combined effect of the 3 heavy metals on urinary NAG levels was significant and stronger in the exposure group than in the control group. However, no relationship was observed between the exposure concentrations of the 3 heavy metals and urinary β2-MG levels. The results suggest that the BKMR model can be used to assess the health effects of heavy-metal exposure on vulnerable residents.

## 1. Introduction

The most common sources of exposure to heavy metals are mines, smelters, and air pollution.^[1]^ Exposure to heavy metals from smelters may cause severe health challenges for nearby residents because the residents are likely to be exposed to multiple and high concentrations of heavy metals^[2]^ (see also Kim and Ryang 2006). The Janghang Refinery in Seocheon, Chungcheongnam-do, Korea, was built in 1936 and was in operation until 1989, mainly smelting copper, lead (Pb), and tin. Investigation of heavy metals in the soil and water around the smelter confirmed contamination with harmful heavy metals such as cadmium (Cd), arsenic (As), Pb, and mercury (Hg).^[3]^

A large-scale cross-sectional study conducted in 2008 revealed that concentrations of various heavy metals, including Pb, Hg, As, and Cd, particularly Cd, were higher in residents living near smelters than in control individuals.^[4]^ Moreover, significant changes were observed in renal tubular damage markers and bone mineral density indicators.^[5]^ However, most studies have focused on the health effects of exposure to individual heavy metals. Urinary Cd, As, and Hg levels are associated with kidney damage, particularly tubular damage.^[6,7]^ Therefore, for multiple exposures to such heavy metals, their combined rather than individual health effects must be evaluated.

Generally, the health effects of multiple exposures to hazardous chemicals are evaluated using multiple linear regression models that correct for the confounding effects of mixed exposure and estimate the independent effects of each hazardous chemical.^[8–10]^ However, when multiple metals have combined effects due to interactions, collinearity owing to the correlation between them is likely to occur, and such combined effects are unlikely to be synergistic or nonlinear. Therefore, it is necessary to quantify the synergistic effects of different metals on disease risk using a mixture analysis. The use of the Bayesian Kernel Machine Regression (BKMR) model, which is a novel statistical method that compensates for the shortcomings above, has been increasing gradually.^[11–13]^

The aim of the present study was to investigate the synergistic effects of combined urinary levels of As, Cd, and Hg on renal damage markers in residents living near smelter plants using the BKMR model. The findings of the present study could highlight the utility of the BKMR model in assessment of the health effects of exposure to multiple heavy metals.

## 2. Materials and methods

### 2.1. Study participants

The study design and methods used to select the study population have been described previously.^[4]^ We recruited 985 study participants living near the Janghang refinery plant and the control area between May and August 2008.

Out of the 985 study participants, 114 (11.6%) with missing data were excluded and 871 were included in the analysis (exposed group: 498, control group: 373). Participants were informed about the objective of the study, and written informed consent was obtained from those who participated. Morning spot urine samples were collected from the participants and maintained at −80°C for preservation until they were analyzed.

### 2.2. Determining toxic metal levels in urine

We measured the levels of Cd, As, and Hg in urine. The methods used for analyzing heavy metals are detailed in our previous research.^[4]^ Cd concentration was determined using a flameless atomic absorption spectrophotometer (Hitachi Model Z-8270), which was outfitted with a Zeeman graphite furnace. Urine samples were combined with nitric acid, diluted using diammonium hydrogen phosphate and 1% Triton X-100, and then mixed thoroughly. The detection limit for Cd was 0.01 µg/L. Total As concentration in urine was analyzed using an atomic absorption spectrometer (PerkinElmer Model 5100) that incorporated a hydride generation system (PerkinElmer FIAS-400). Each urine sample was mixed with HCl, ascorbic acid, and potassium iodide (2:2:1:1); left to incubate for an hour; and then diluted with 10% HCl. The reducing agents used were 0.2% sodium borohydride and 0.5% sodium hydroxide. The mobile phase consisted of 3% HCl, with argon utilized as the carrier gas. The detection limit of the method was 0.2 µg/L. Urine Hg concentration was analyzed using the gold amalgam method with a direct Hg analyzer. After placing 100 µL of well-mixed urine in the sample container, the analysis was conducted immediately.

### 2.3. Determining N-acetyl-β-D-glucosaminidase and β2-microglobulin activities

N-acetyl-β-D-glucosaminidase (NAG) activity was assessed by hydrolyzing sodium m-cresolsulfonephthaleinyl N-acetyl-β-D-glucosaminide to N-acetyl-β-D-glucosaminide and m-cresolsulfonephthalein using NAG. Quantitative analysis of NAG activity was conducted using a commercially available kit (Shionogi, Osaka, Japan), following the manufacturer instructions. Briefly, the synthetic substrate solution (1 mL) was warmed at 37°C for 5 minutes. The urine sample supernatant (50 mL), acquired through centrifugation, was combined with the heated synthetic substrate solution and incubated in a 37°C water bath for 15 minutes. Subsequently, a stopping solution (2 mL) was introduced and mixed thoroughly. The absorbances of the sample and the standard NAG solution were determined at a wavelength of 580 nm via a spectrophotometer.

The urine level of β2-microglobulin (β2-MG) was ascertained using a commercially available kit (Enzygnost β2-MG Micro Kit; Behring Institute, Mannheim, Germany), according to the manufacturer instructions. The kit employs a solid-phase enzyme-linked immunosorbent assay, using a monoclonal anti-β2-MG antibody for immobilization and an anti-β2-MG horseradish peroxidase conjugate solution. The color intensity, which directly corresponds to the concentration of β2-MG, was measured spectrophotometrically at 450 nm. Urine creatinine levels (g/L) were measured using the Jaffe method.

### 2.4. Statistical analysis

The distributions of urinary Cd, As, Hg, and renal tubular damage markers had severe right-skewness; therefore, they were converted into a natural logarithmic form and applied to the regression model. Age, sex, drinking and smoking status, monthly household income, and urinary creatinine concentration were included as covariates in the regression model to control for confounding variables. Multiple linear regression models were used to evaluate the associations between heavy metals and renal tubular damage markers. The multiple linear regression equation was as follows:

$$\begin{array}{l} Yi=\beta 0+\beta 1Cdi+\beta 2Asi+\beta 3Hgi+\beta TZi+ei \end{array}$$ (1)

where Y is the log-transformed renal tubular damage marker level; Cd, As, and Hg are the centered log concentrations of As, Cd, and Hg, respectively; and Z = Z1,..., Zp are additional potential confounders including age, sex, smoking, drinking habits, and economic status.

The BKMR analysis was performed to estimate the combined effects of exposure to the 3 heavy metals and their nonlinear effects of heavy metals.^[11]^ The BKMR model is expressed as follows:

$$\begin{array}{l} Yi=h\left(Cdi,\;Asi,\;Hgi\right)+\beta TZi+ei \end{array}$$ (2)

We implemented 10,000 iterations of the Markov chain Monte Carlo algorithm.^[14]^ Generally, in environmental mixtures, h(·) depicts a high dimensional exposure − response function that could involve nonlinear relationships or interactions among the components of the mixture. To relax the linearity and additive effect assumptions required in the regression model, a BKMR model was applied to estimate the joint exposure–response functions of the 3 urinary heavy metals. Estimates of the mixed exposure effects were obtained by calculating the post-estimate means and 95% confidence intervals (CIs) of renal tubular damage markers associated with changes in the level of exposure to each heavy metal. Regarding the combined effect, the expected change in renal tubular damage markers associated with the simultaneous change in the 3 heavy metals was estimated and compared with the median exposure level to the heavy metal mixture. Additionally, with the concentrations of the other 2 heavy metals set to the 25th, 50th, and 75th percentile values, the expected change in renal tubular damage markers according to the interquartile range (IQR) change in the concentration of each heavy metal was estimated. Finally, the dose-response relationship of each mixed component and potential interactions between metals were evaluated, with the concentrations of other heavy metals fixed at the 25th, 50th, and 75th percentiles. All statistical analyses were performed using R software (version 4.2.3; R Foundation for Statistical Computing). Statistical significance was set at *P* < .05 significant.

### 2.5. Ethical statement

The study protocol was approved by the Institutional Review Board of Chungbuk National University (CBNU-IRB-2011-BQ02), and all the participants provided written informed consent.

## 3. Results

### 3.1. Study population characteristics

**Figure 1.**
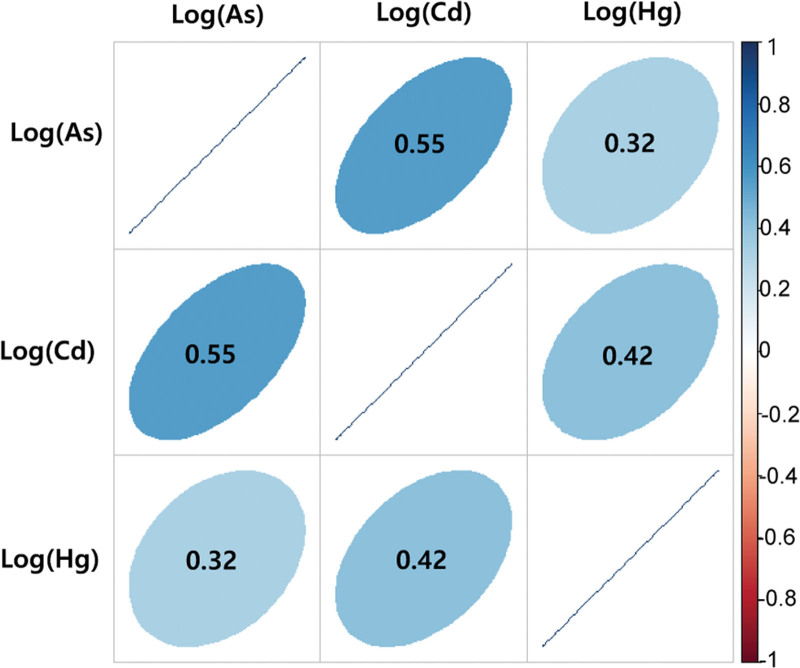
Pearson correlation matrix for log-transformed urinary heavy metals.

The demographic characteristics of the study participants are summarized in Table 1. Among the participants, 41.33% (n = 360) were men, and 15.15% (n = 132) were smokers. A total of 694 participants (79.68%) had an average monthly income of less than 1 million won. The average age of the study participants was 64.14 years, with that of the control participants being relatively higher than that of the exposed group. The geometric mean concentrations of urinary Cd, As, Hg, and NAG were higher in the exposed group than in the control group. Pearson correlation coefficients between the log-transformed urinary heavy metal concentrations were 0.55 for Cd and As, 0.42 for Hg and Cd, and 0.32 for urinary Hg and As (Fig. 1).

**Table 1:** Demographic characteristics of study participants.

	Total (n = 871)	Exposed group (n = 498)	Control group (n = 373)
Sex, n (%)			
Male	360 (41.33)	217 (43.57)	143 (38.34)
Female	511 (58.67)	281 (56.43)	230 (61.66)
Smoking status, n (%)			
Nonsmoker	739 (84.85)	413 (82.93)	326 (87.40)
Smoker	132 (15.15)	85 (17.07)	47 (12.60)
Drinking status, n (%)			
Nondrinker	443 (50.86)	243 (48.80)	200 (53.62)
Drinker	428 (49.14)	255 (51.20)	173 (46.38)
Economic status, n (%)			
High	177 (20.32)	126 (25.30)	51 (13.67)
Low	694 (79.68)	372 (74.70)	322 (86.33)
Age, yr, Mean (SD)	64.14 (11.42)	63.12 (11.33)	65.51 (11.42)
Urinary As, µg/L, GM (GSD)	8.01 (1.87)	8.35 (1.87)	7.57 (1.88)
Urinary Cd, µg/L, GM (GSD)	2.02 (2.50)	2.51 (2.33)	1.50 (2.53)
Urinary Hg, µg/L, GM (GSD)	0.46 (6.11)	0.51 (5.54)	0.39 (6.85)
NAG, unit/L, GM (GSD)	2.55 (5.14)	2.83 (4.45)	2.23 (6.08)
β2-MG, mg/L, GM (GSD)	0.02 (12.22)	0.02 (11.88)	0.02 (12.71)

As = arsenic, Cd = cadmium, GM = geometric means, Hg = mercury, NAG = N-acetyl-β-D-glucosaminidase, SD = standard deviation, β_2_-MG = β_2_-microglobulin.

### 3.2. Multivariate regression analyses

Table 2 presents the relationship between urine heavy metal concentrations and renal tubular damage markers using multivariate linear regression models adjusted for confounding factors. In all participants, with increasing urine Cd concentration, NAG concentration increased significantly (β:0.447, 95% CI: 0.290–0.603). However, the concentrations of other heavy metals had no significant effect on urine NAG concentration. A similar trend was observed in the stratification analysis of groups. However, the regression coefficients in the exposed and control groups were 0.458 and 0.358, respectively, indicating relatively large regression coefficients in the exposed group.

**Table 2:** Association between urinary As, Cd, and Hg levels and renal damage biomarkers, adjusted for sex, age, lifestyle factors, economic status, and urinary creatinine level: A multivariate ordinary least squares regression analysis.

	β (95% CI)	
	NAG	β2-MG
Total		
As	−0.053 (−0.271, 0.165)	0.098 (−0.266, 0.463)
Cd	0.447 (0.290, 0.603)	0.061 (−0.199, 0.323)
Hg	−0.002 (−0.065, 0.062)	−0.265 (−0.133, 0.080)
Exposed group		
As	0.048 (−0.215, 0.310)	0.184 (−0.303, 0.671)
Cd	0.458 (0.255, 0.661)	−0.010 (−0.388, 0.367)
Hg	0.016 (−0.062, 0.063)	−0.027 (−0.171, 0.118)
Control group		
As	−0.118 (−0.493, 0.257)	0.044 (−0.522, 0.609)
Cd	0.358 (0.062, 0.654)	0.072 (−0.374, 0.518)
Hg	−0.010 (−0.114, 0.095)	−0.014 (−0.172, 0.144)

As = arsenic, Cd = cadmium, Hg = mercury, NAG = N-acetyl-β-D-glucosaminidase, β2-MG = β2-microglobulin.

### 3.3. Bayesian Kernel Machine Regression analysis

**Figure 2.**
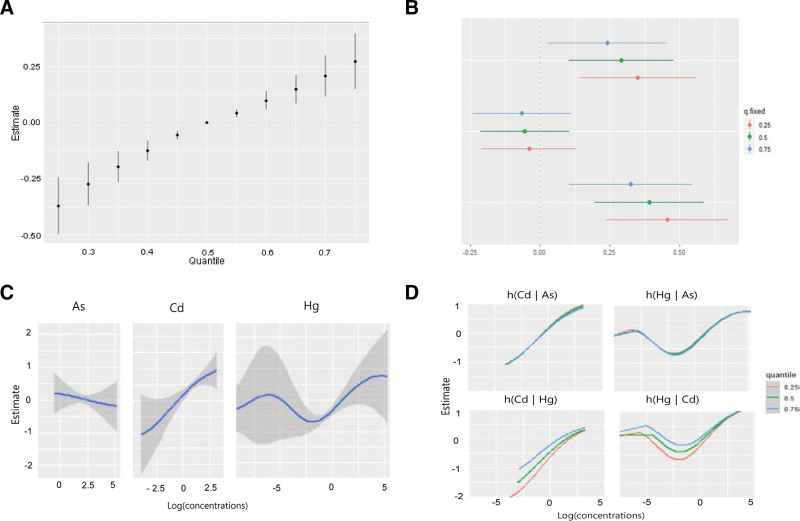
The combined effect of the heavy metals on urine NAG concentrations using the Bayesian Kernel Machine Regression (BKMR) model. The model was adjusted for age, sex, drinking, smoking, economic status, and urinary creatinine level. (A) The overall impact of combined metal exposures (estimates and 95% confidence intervals for the h function). This figure depicts the estimated shifts in urine NAG levels when exposure is at a certain percentile (x-axis) versus exposure at the 50th percentile. (B) Single pollutant associations (estimates and 95% confidence intervals, with a gray dashed line representing the null). This plot shows NAG level variations when a single pollutant is at the 75th percentile compared to the 25th percentile, while maintaining other exposures at the 25th, 50th, or 75th percentile. (C) Univariate exposure − response curves and 95% confidence bands for each metal, while maintaining other pollutants at the median. (D) Bivariate exposure − response charts for cadmium: when arsenic is set at the 25th, 50th, or 75th percentile and mercury at the median (top left); when arsenic is at the 25th, 50th, or 75th percentile and cadmium is at the median (top right); when mercury is at the 25th, 50th, or 75th percentile and arsenic is at the median (bottom left); and when cadmium is at the 25th, 50th, or 75th percentile and arsenic is at the median (bottom right). NAG = N-acetyl-β-D-glucosaminidase.

We estimated the change in urine NAG levels when the concentrations of the 3 heavy metals changed to a specific critical value (25th–75th percentile) compared with the median concentration of each heavy metal. The combined effects of the 3 heavy metals were significantly associated with urinary NAG levels below and above the 45th and 55th percentiles, respectively (Fig. 2A). We assessed which of the 3 metals significantly affected NAG concentrations by estimating the univariate summaries of the changes in urinary NAG levels associated with IQR changes in single heavy metals. Urine Cd levels had the greatest effect on NAG levels, and Hg levels significantly affected NAG levels. Furthermore, urine Hg and Cd levels had the greatest effect on NAG levels when the concentration of other heavy metals was at the 25th percentile, compared with the 50th and 75th percentiles. When the urine As and Cd concentrations were fixed at the 25th, 50th, and 75th percentiles, the urine NAG levels based on the IQR increase in urine Hg levels were 0.351 (95% CI: 0.142–0.560), 0.291 (95% CI: 0.103–0.480), and 0.242 (95% CI: 0.029–0.455), respectively. When urine Hg and Cd concentrations were fixed at the 25th, 50th, and 75th percentiles, urine NAG levels based on the IQR increase in urine As were −0.039 (95% CI: −0.209, 0.131), −0.055 (95% CI: −0.215, 0.105), and −0.065 (95% CI: −0.240, 0.110), respectively (Fig. 2B). When urine Hg and As concentrations were fixed at the 25th, 50th, and 75th percentiles, the urine NAG levels based on the IQR increase in urine Cd were 0.455 (95% CI: −0.237, 0.674), 0.391 (95% CI: 0.195–0.587), and 0.325 (95% CI: 0.104–0.546), respectively (Fig. 2B).

**Figure 3.**
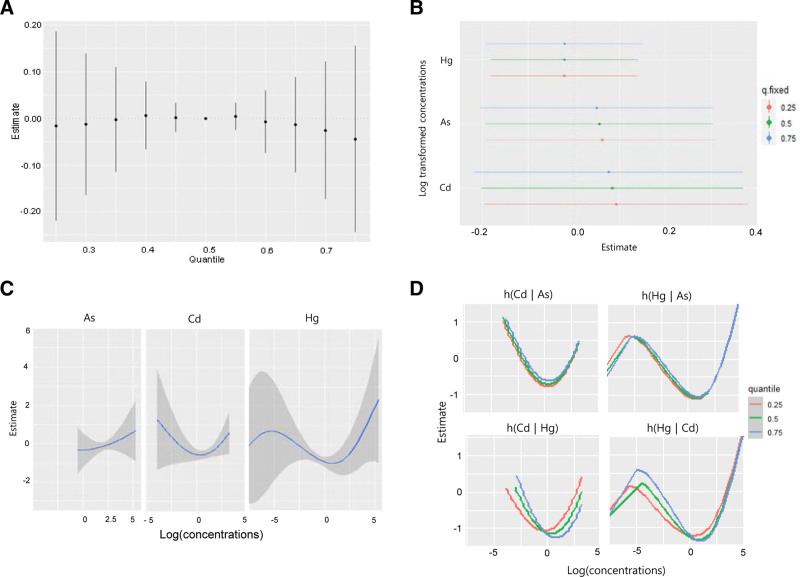
The combined effect of exposure to the mixture of the metals on urinary β2-MG concentration using the Bayesian Kernel Machine Regression (BKMR) model. The model was adjusted for age, sex, drinking, smoking, economic status, and urinary creatinine level. (A) The overall impact of the mixture (estimates and 95% confidence intervals for the h function). This figure depicts the estimated variation in urine β2-MG levels when exposure is at a certain percentile (x-axis) versus exposure at the 50th percentile. (B) Single pollutant associations (estimates and 95% confidence intervals, with a gray dashed line representing the null). This plot shows β2-MG level variations when a single pollutant is at the 75th percentile compared to the 25th percentile, while maintaining other exposures at the 25th, 50th, or 75th percentile. (C) Univariate exposure − response functions and 95% confidence bands for each metal, while maintaining other pollutants at the median. (D) Bivariate exposure − response functions for cadmium: when arsenic is set at the 25th, 50th, or 75th percentile and mercury at the median (top left); when arsenic is at the 25th, 50th, or 75th percentile and cadmium is at the median (top right); when mercury is at the 25th, 50th, or 75th percentile and arsenic is at the median (bottom left); and when cadmium is at the 25th, 50th, or 75th percentile and arsenic is at the median (bottom right). β2-MG = β2-microglobulin.

Furthermore, based on the exposure–response function between each heavy metal and urine NAG concentration when the other 2 metals were fixed at the median, a nonlinear relationship was observed between urine Hg concentration and NAG level. In addition, urinary As levels were negatively associated with NAG levels (Fig. 2C). No interaction existed between As and Cd levels or between As and Hg levels when urine Hg and Cd were fixed at the median levels. However, when urine As was fixed at the median level, Hg and Cd levels exhibited some interactions with urine NAG levels (Fig. 2D). However, when analyzing the association between combined exposure to the 3 heavy metals and the β2-MG urine concentrations, no combined effect was observed (Fig. 3A). No metal significantly affected the change in urinary β2-MG level, which was associated with the change from the 25th to the 75th percentile of a single heavy metal (Fig. 3B). In addition, minimal interactions were observed between the metals that affected the urine β2-MG concentration changes (Fig. 3C and D).

**Figure 4.**
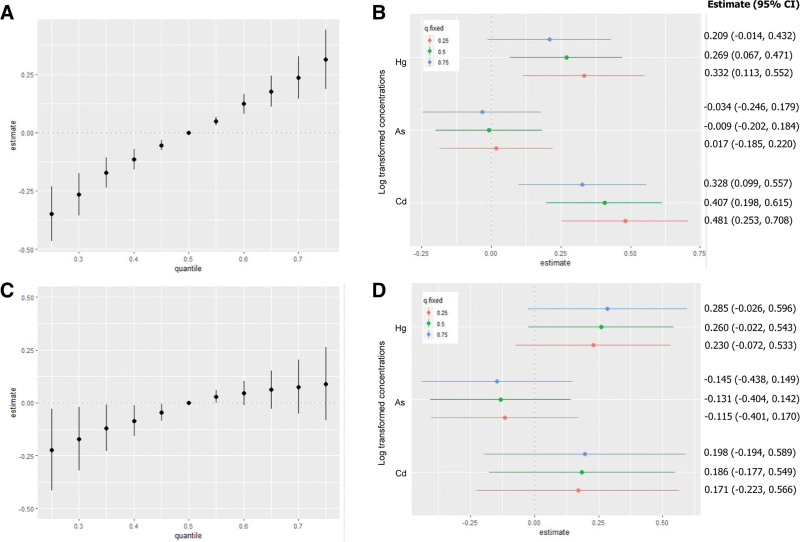
The combined effect of the mixture on urinary NAG concentration using the Bayesian Kernel Machine Regression (BKMR) model. The model was adjusted for age, sex, drinking, smoking, economic statuses, and urinary creatinine level in the exposed (A and B) or control group (C and D). (A and C) The cumulative impact of the compound (estimates and 95% confidence intervals for the h function). This graph illustrates the estimated alteration in urinary NAG levels when exposures are situated at a specific percentile (x-axis) in contrast to when all exposures are at the 50th percentile. This figure presents the cumulative impact of the mixture (estimates and 95% confidence intervals for the h function). It demonstrates the estimated alteration in urinary NAG levels with exposures at specific percentiles (x-axis), compared to all exposures being at the 50th percentile. (B and D) Associations with individual pollutants are shown (estimates and 95% confidence intervals, with a gray dashed line indicating the null). This illustration contrasts NAG levels when a single pollutant is at the 75th percentile against the 25th percentile, while maintaining all other exposures constant at the 25th, 50th, or 75th percentile. NAG = N-acetyl-β-D-glucosaminidase.

A stratified analysis was conducted to determine the relationship between heavy metal exposure and urine NAG concentration. In the exposed group, the combined effects in the 25th and 75th percentiles were −0.349 and 0.314, respectively, compared with the median concentration of each heavy metal (Fig. 4A). When the other 2 metals were set at the 25th, 50th, and 75th percentiles, urine Hg and Cd levels were significantly and positively associated with urine NAG levels. When urine As and Cd concentrations were fixed at the 25th, 50th, and 75th percentiles, the urine NAG levels based on the IQR increase in urinary Hg were 0.332 (95% CI: 0.113–0.552), 0.269 (95% CI: 0.067–0.471), and 0.209 (95% CI: −0.014, 0.432), respectively. When urinary Hg and As concentrations were fixed at the 25th, 50th, and 75th percentiles, urine NAG levels based on the IQR increase in urinary Cd were 0.481 (95% CI: 0.253–0.708), 0.407 (95% CI: 0.198–0.615), and 0.328 (95% CI: 0.099–0.557), respectively (Fig. 4B). In contrast, in the control group, the estimated combined effects at the 25th and 75th percentile were −0.223 and 0.090, respectively, compared with the median concentration of each heavy metal. Moreover, at concentrations above the 60th percentile, no significant changes were observed in the combined effect of heavy metals on the NAG concentration (Fig. 4C). When the other 2 metals were set at the 25th, 50th, and 75th percentiles, no metals were associated with urinary NAG levels (Fig. 4D).

## 4. Discussion

This study used the BKMR model to investigate the effects of combined exposure to Cd, As, and Hg, which are associated with renal damage, on NAG and β2-MG (representative renal tubular damage markers) in residents living near smelters. The BKMR model is increasingly used in environmental epidemiology because it overcomes the nonlinear relationship between hazardous chemicals and health effects and potential interactions between chemicals and can evaluate the combined effects of chemical mixtures.^[11]^ Previous studies have reported a relationship between heavy metal exposure and health effects in residents near smelters.^[15–17]^ Residents living near smelters have significantly higher exposure to Cd, Hg, Pb, As, and Cu, and there is a relationship between exposure to these heavy metals and kidney damage.^[4,5,8]^ NAG and β2-MG are markers used to evaluate renal tubular damage.^[6,7]^ Cd can directly affect renal tubular cells, causing cell damage and inflammation. After cell damage, the excretion of proteins synthesized in the tubules may increase or their reuptake might be inhibited.^[18]^ Lim et al^[19]^ investigated the impacts of low-level exposure to Pb and Cd in a large cross-sectional study on a Korean adult population. They observed positive correlations between the urinary concentrations of Pb and Cd and NAG and β2-MG levels in urine. An interactive effect of Pb and Cd exposure on urinary NAG and β2-MG levels was also observed. This study underscores the significance of exposure to multiple heavy metals, even at low levels.

Mercury toxicity is a significant global health challenge (Branco et al^[20]^; UN Environment Global Mercury Assessment, 2018). Proximal tubular damage after exposure to high Hg concentrations is related to oxidative stress due to depletion of the cellular thiol pool.^[21]^ Increased urinary NAG activity was reported in workers exposed to low Hg concentrations for extended periods.^[22–24]^ However, studies on the relationship between amalgam fillings, a major source of Hg exposure, and urinary NAG levels are diverse. Studies have revealed that dental amalgam levels positively correlated with urine NAG levels.^[25,26]^ In contrast, some studies have reported that amalgam filling in children has no relationship with NAG levels,^[27,28]^ and that no difference exists in the renal function of patients before and after amalgam filling.^[29,30]^

As is one of the most common hazardous substances in water and soil.^[31]^ Exposure to As in drinking water causes renal damage and chronic kidney disease.^[21]^ In addition, chronic As exposure increases urine NAG levels in individuals living in As-contaminated areas.^[32]^ Renal tubular damage is aggravated when cells are exposed to Cd and As for extended periods, even at low concentrations.^[33]^

Unlike workers who are exposed to specific heavy metals, most of the general population is likely to be exposed to multiple combinations of harmful heavy metals. The relationship between exposure to heavy metals and health effects differs in various epidemiological studies, possibly because the combined exposure to multiple heavy metals may cause different health effects. Depending on the interactions between heavy metals, the relationship between heavy metals and health effects may be linear or nonlinear. Therefore, examining the relationship between a single heavy metal and its health effects using only a linear regression model cannot rule out the possibility of distorted results.

In this study, we evaluated the relationships between urinary As, Cd, and Hg concentrations and renal tubular damage markers using a multiple linear regression model. Urinary Cd was significantly correlated with NAG concentration. However, the differences in As and Hg levels were not significant. In addition, in the multiple regression analysis with β2-MG concentration as the dependent variable, no significant differences were observed for any of the 3 heavy metals. In contrast, in the BKMR model, when the effects of each heavy metal were evaluated with other heavy metals at constant levels, the concentrations of urinary Hg and Cd significantly affected the changes in NAG levels.

The difference in the effect of urinary Hg on NAG levels in the BKMR and multiple linear regression models was most likely due to their nonlinear relationship (Fig. 2C). A multiple linear regression model evaluates the linear relationship between 2 variables; therefore, statistical significance may not appear in a nonlinear relationship.

In addition, the effects of combined exposure to heavy metals may vary depending on the degree of exposure. In this study, the stratified BKMR analysis of the relationship between heavy metal exposure and urine NAG concentration revealed that the combined effect in the exposed group was greater than that in the control group.

Most mixed analysis methods exhibit certain limitations. For example, the clustering approach groups mixtures into different subgroups, which can cause information loss. Therefore, it is unsuitable for downstream analysis,^[34]^ and shrinkage methods such as LASSO penalize less important variables as “0.” Moreover, it is typically used for linear exposure–response functions; therefore, it is limited in explaining the combined effects of heavy metals on health.^[35]^ In this regard, BKMR can measure the overall mixed effects and separately evaluate the effects of individual predictors and confounding variables in a nonlinear and nonadditive manner.^[11]^

Nevertheless, this study has several limitations. First, this study used a cross-sectional design; thus, identifying the causal relationship between heavy metals and health effects is challenging. Second, only 3 heavy metals in urine (As, Cd, and Hg) were included in the analysis; however, residents living near the smelters are exposed to more heavy metals. This is because we included only heavy metals measured in the urine rather than those measured in the blood. Finally, the total As and total Hg concentrations were used in the study, although the toxicity of As and Hg to the kidney varies based on their chemical properties. In the future, the effects of inorganic As and organic Hg, which are known to be highly toxic, on the kidney, need to be investigated.

In summary, we evaluated the effects of combined exposure to As, Cd, and Hg on urine NAG levels in residents living near smelters using a BKMR model. The combined effects of the 3 heavy metals were significantly associated with increased urine NAG concentrations, which were caused by increased urine Cd and Hg concentrations. This association was more evident in the individuals exposed to high concentrations of heavy metals. These results suggest that the BKMR model is useful for assessing the health effects of heavy metal exposure.

## 5. Conclusions

This study used a BKMR model to assess the effect of combined exposure to As, Cd, and Hg on the renal health of individuals living near smelters. Exposure to these heavy metals significantly increased urinary NAG levels, a renal tubular damage marker, primarily because of elevated levels of Cd and Hg. This relationship was notably stronger in individuals exposed to high concentrations, indicating the utility of the BKMR model for assessing the health effects of multiple heavy metals.

## Author contributions

**Conceptualization:** Sun-Haeng Choi, Jong-Uk Won, Heon Kim.

**Formal analysis:** Sun-Haeng Choi, Kyung Hi Choi.

**Funding acquisition:** Heon Kim.

**Investigation:** Sun-Haeng Choi, Heon Kim.

**Methodology:** Kyung Hi Choi.

**Supervision:** Jong-Uk Won, Heon Kim.

**Validation:** Jong-Uk Won.

**Visualization:** Sun-Haeng Choi.

**Writing – original draft:** Sun-Haeng Choi.

**Writing – review & editing:** Jong-Uk Won, Heon Kim.
